# A path analytic model of health beliefs on the behavioral adoption of breast self-examination

**DOI:** 10.3934/publichealth.2021002

**Published:** 2020-12-21

**Authors:** Soo-Foon Moey, Norfariha Che Mohamed, Bee-Chiu Lim

**Affiliations:** 1 Department of Diagnostic Imaging and Radiotherapy, Kulliyyah of Allied Health Sciences, International Islamic University Malaysia (IIUM), Kuantan Campus, Pahang, Malaysia; 2 Clinical Research Centre, Hospital Tengku Ampuan Afzan (HTAA), Kuantan, Pahang, Malaysia

**Keywords:** Health belief model, behavioral adoption, breast self-examination, breast cancer knowledge, structural equation modeling

## Abstract

**Background:**

In Malaysia, breast cancer accounted for 34.1% of all female cancer cases with women presenting breast cancer at late stages. Breast cancer has a higher five-year survival rate if detected early. An increase of approximately 30% in the five-year survival rate is indicated if breast cancer is detected at stage III compared to stage IV. Thus, survival rate of breast cancer can be increased by creating awareness and encouraging breast cancer screening amongst women. Breast self-examination (BSE) is highly recommended for breast cancer screening due to its simplicity with no incurred cost. The Health Belief Model is used in this study to explain and predict the adoptive behavior of BSE amongst women in Kuantan, Pahang.

**Materials and methods:**

This study employed a multi-stage sampling method using a simple proportion formula at 5% type 1 error, *p* < 0.05 and absolute error at 2% which resulted in a sample of 520 participants. The data for the study was obtained using a validated bilingual self-constructed questionnaire and the model constructed using Mplus software.

**Results:**

Perceived severity, benefits and barriers were found to significantly influence the behavioral adoption of BSE. Married women aged from 45 to 55 years and knowledge were found to significantly moderate the relationship between perceived benefits and behavioral adoption of BSE. Further, self-efficacy was found as the core construct that mediates the relationship between married women aged 45 to 55 years and the behavioral adoption of BSE.

**Conclusion:**

Self-efficacy is found in the study to influence the behavioral adoption of BSE. This is undeniable as self-efficacy can promote confidence in initiating and maintenance of behavioral change if the perceived change is beneficial at an acceptable cost.

## 1. Introduction

Breast cancer is the most common cancer amongst women globally accounting for 25.4% of total new cases in 2018 [1]. In Malaysia, breast cancer is the most frequent cancer amongst women accounting for 34.1% of all female cancer cases. A total of 21,634 breast cancer cases were diagnosed from 2012 to 2016 compared with only 18,206 cases diagnosed from 2007 to 2011 [2]. Besides, Malaysia has the highest mortality rate accounting for 18 per 100,000 compared to Singapore and Thailand which is only 15 and 11 per 100,000 populations respectively [3]. Further, Malaysian women presented at later stages compared to women in Singapore and the western countries which affected the survival rate. Heterogeneous findings were reported from various studies on the overall median survival time of breast cancer in Malaysia. In a population-based study, an overall median survival time of 68.1 months was reported [4]. However, another study revealed a shorter median survival time of 54 months [5]. Further, using data from the Singapore-Malaysia Breast Cancer Registry, a median survival time of 164 months was reported for stage II. However, for stage III and stage IV, the median survival time was reduced to 53 months and 17 months respectively [6]. The findings reflected that as the stage of breast cancer increased, the median survival time will be reduced drastically.

The morbidity and mortality rate can be reduced to improve the survival rate of breast cancer patients by creating awareness and encouraging breast cancer screening amongst Malaysian women [7]. One of the highly recommended screening methods is breast self-examination (BSE) due to its simplicity, no cost and easy to learn without any sophisticated technology [8]. However, the uptake of BSE amongst Malaysian women remains low as reported in previous studies [9]. In a suburban district in Selangor, studies showed that only 58.5% of women practiced BSE while in urban areas only 47.2% of women practiced BSE on a monthly basis. Additionally, in a study conducted amongst female undergraduate students at University Putra Malaysia, only 36.7% of them were reported performing BSE [10]–[12].

Realizing breast cancer as one of the health priorities in Malaysia, women should be empowered to perform BSE through more effective educational and training programs based on theory-driven approaches [13]. The health belief model (HBM) is the most widely used model or theory which conceptualizes potential barriers or factors that influence a desired health behavioral adoption [14]. It is thus recommended to employ the health behavior theories in programs and interventions using derived theoretical constructs and pathways [15],[16] to enable drive behavior changes. In order to conceptualize HBM constructs comprehensively, this study analyzed the direct and indirect causal pathways that influence the behavioral adoption of BSE. Further, the moderating effects of socio-demographic factors and knowledge and also the mediating effects of self-efficacy were included to predict the variance in the adoptive behavior of BSE using structural equation modeling fit statistics [16]. It is believed that the model obtained from this study can be used to develop intervention and training programs amongst women in Kuantan, Pahang to increase awareness and improve their BSE performance.

## 2. Materials and method

### 2.1. Study design and participants

This cross-sectional study was carried out to ascertain the relationship between health belief constructs and behavioral adoption of BSE, moderated by socio-demographic factors and knowledge pertaining to breast cancer screening. This study further hypothesized that self-efficacy mediates the relationship between socio-demographic factors and knowledge on the behavioral adoption of BSE. This study took place between February and April of 2018 in three sub-districts in Kuantan, Pahang. Pahang is the third largest state in Malaysia by area and ninth largest by population. Pahang is situated on the east coast of West Malaysia with a 50% urban population. Kuantan is the capital of Pahang and the eighth largest urban area by population in Malaysia [17]. The sample size was acquired using cluster random sampling followed by stratified random sampling. The largest polyclinic in the three sub-districts namely Kuala Kuantan, Sungai Karang and Beserah were selected using this method. The target population for the study sample was women aged between 35 to 70 years able to read and write in Bahasa Malaysia or English and living in Kuantan. By employing a simple proportion formula at 5% type 1 error, *p* < 0.05 and absolute error at 2%, a sample size of 520-subjects was obtained.

### 2.2. Data collection

Before conducting the study, permission and approval were obtained from the Medical Research and Ethics Committee (MREC) (NMRR-17-2131-37586 (IIR)), International Islamic University Malaysia Research Ethics Committee (IREC) (IREC 2017-075) and the Kulliyyah Postgraduate Research Center (KPGRC) (KAHS 173). Potential participants were women that accompany their relatives or friends to the polyclinics. An information sheet was given to them to clarify the purpose of the study. Verbal consent was obtained prior to the questionnaires being administered.

### 2.3. Instrument

The instrument is a bilingual self-constructed questionnaire comprising of four sections. The first section is pertaining to socio-demographic factors (age, race, marital status, level of education, occupation and family income) whilst the second section pertaining to knowledge on breast cancer screening and the third section is regarding the participants' health beliefs of BSE. In the fourth section, the final section is to solicit the behavioral adoption of BSE amongst participants. The questionnaire was self-developed and the content validated by an English lecturer and four health professionals; a radiologist specializing in breast imaging, a research scholar in women's health and two professors in the related health field. Prior to data collection, the questionnaire was pilot-tested for validity and reliability using exploratory factor analysis (EFA) [14].

### 2.4. Data analysis

The constructs derived from the Health Belief Model; perceived susceptibility, perceived severity, perceived benefits, perceived barriers and motivation factors were operationalized as independent variables and the behavioral adoption of BSE as the dependent variable. Knowledge and socio-demographic factors were hypothesized to moderate the relationship of the fore mentioned health beliefs (independent variables) and the behavioral adoption of BSE (dependent variable). Self-efficacy was also hypothesized to mediate the relationship between knowledge and socio-demographic factors and behavioral adoption of BSE. A 10-point Likert scale was used to assess perceived susceptibility, perceived severity, perceived benefits, perceived barriers, motivation factors, self-efficacy and cues to action. The four cues to action (cues to action 1 to 4), see Appendix 1.

Structural equation modeling (SEM) was used to validate the relationships between health belief constructs and behavioral adoption of BSE while controlling for moderating effects of socio-demographic factors and knowledge pertaining to breast cancer screening as well as the mediating effects of self-efficacy. SEM is a statistical method that involves a confirmatory approach for analyzing a structural theory on a particular phenomenon [18]. In this study, the Mplus software program version 8.3 using maximum likelihood with robust standard errors (MLR) estimator was employed to perform the SEM. Re-specification or modification of a model was used in the study as it is a common practice in structural equation modelling. Modification is necessary for all SEM models because they rarely pass the model fit test when compared with the set of data in the initial stage.

To evaluate model fitness for SEM, a number of fit indices were taken into comprehensive consideration. The fit indices used in this study included the Comparative Fit Index (CFI) and Tucker Lewis Index (TLI) values, of which a value of 0.90 or greater indicate a good fit. The Standardized Root Means Square Residual (SRMR) value less than or equal to 0.08 and the Root mean square error of approximation (RMSEA) value of 0.08 or less indicate a reasonable fit [19]–[22]. Subsequent path models were re-examined and modified to obtain a better fitting model if modification indices (MI) were larger than 10 [23],[24]. Modification was applied when there is a theoretical justification for them [25],[26]. Significant standardized path coefficients (β) with 95% confidence interval (CI), standard error (SE) and statistical significance value were reported for the finalized path model. Significance level was set at α = 0.05.

**Figure 1.**
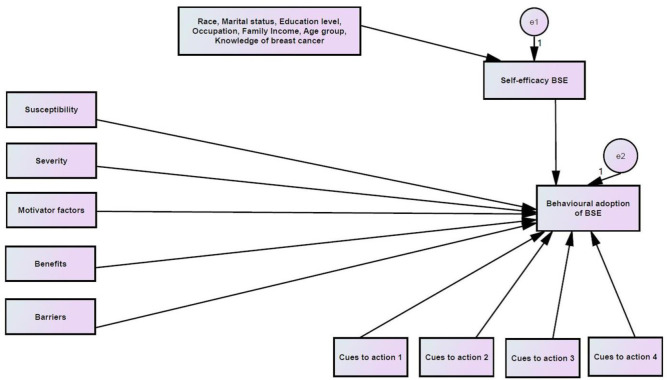
Initial path model (Model 1) represents the hypothesized relationships between constructs that influence BSE behavioral adoption. Note: e1–e2 are the errors in measurement.

The initial path model represents the hypothesized relationships between constructs that influence BSE behavioral adoption. The specific hypotheses for each path relationships are illustrated in Figure 1.

The initial model (Figure 1) represents the path relationships between socio-demographic factors (age, race, marital status, education level, family income and occupation), knowledge pertaining to breast cancer, health beliefs constructs (perceived susceptibility, severity, benefits, barriers, self-efficacy, motivator factors and cues to action) and the behavioral adoption of BSE. There were 17 hypothesized path relationships for the initial model (Model 1) of which socio-demographic characteristics and HBM constructs were also considered. Table 1 illustrates the hypotheses postulated for the initial model (Model 1).

**Table 1.** Hypotheses for Model 1.

Hypotheses	
H1:	Race significantly influence self-efficacy.
H2:	Marital status significantly influences self-efficacy.
H3:	Education level significantly influence self-efficacy.
H4:	Occupation significantly influence self-efficacy.
H5:	Family income significantly influence self-efficacy.
H6:	Age significantly influence self-efficacy.
H7:	Knowledge of breast cancer significantly influence self-efficacy.
H8:	Self-efficacy significantly influences behavioral adoption of BSE.
H9:	Perceived susceptibility significantly influences behavioral adoption of BSE.
H10:	Perceived severity significantly influences behavioral adoption of BSE.
H11:	Motivator factors significantly influence behavioral adoption of BSE.
H12:	Perceived benefits significantly influence behavioral adoption of BSE.
H13:	Perceived barriers significantly influence behavioral adoption of BSE.
H14:	Cues to action 1 (those who had heard about BSE from the mass media) significantly influence behavioral adoption of BSE.
H15:	Cues to action 2 (those who had heard about BSE from friends/partner/doctor/healthcare provider) significantly influence behavioral adoption of BSE.
H16:	Cues to action 3 (those who known someone who had breast cancer) significantly influence behavioral adoption of BSE.
H17:	Cues to action 4 (those having a close relative who had breast cancer) significantly influence behavioral adoption of BSE.

Note: H = hypothesis.

Based on the conceptual framework of the study, we assume that self-efficacy would mediate the relationships between socio-demographic factors and knowledge on breast cancer and behavioral adoption of BSE. The indirect effects testing using model indirect command in the Mplus program was conducted to test whether self-efficacy mediates the relationships as suggested by Wang and Wang [24]. A *p*-value < 0.05 was considered a significant indirect path.

The possible moderating effects of socio-demographic factors on individual health belief constructs influencing the behavioral adoption of BSE were also carried out. Biological plausible and important interaction terms between significant socio-demographic factors and individual health belief constructs that influence the behavioral adoption of BSE were created. The interaction terms were iteratively included in the path model using Mplus and the level of significance evaluated for the interaction terms. A *p*-value < 0.05 in the moderation path was considered as statistically significant.

## 3. Results

The socio-demographic characteristics of the participants involved in this study are as shown in Table 2 and the summary of fit indices of the path models are as in Table 3.

**Table 2.** Socio-demographic characteristics of participants.

Socio-demographic characteristics	Frequency (%)
Age (years)	
35–45	322 (61.9)
46–55	121 (23.3)
56 and above	77 (14.8)
Race	
Non-Malay	33 (6.3)
Malay	487 (93.7)
Marital status	
Single	106 (20.4)
Married	414 (79.6)
Education level	
Higher education	273 (52.5)
Secondary school	214 (41.2)
No formal education to primary school	33 (6.3)
Occupation	
Private and self-employed	99 (19.0)
Government staff	268 (51.5)
Part-timer	153 (29.4)
Family income (RM)	
6,000 to >10,000 (high income)	34 (6.5)
3,000 to 5,999 (middle income)	253 (48.7)
<1,000 to 2,999 (low income)	233 (46.8)
Heard about BSE from the mass media (Cues 1)	
No	64 (12.3)
Yes	456 (87.7)
Heard about BSE from friends/partner/doctor/healthcare provider (Cues 2)	
No	58 (11.2)
Yes	462 (88.8)
Known someone who had breast cancer made me do BSE (Cues 3)	
No	193 (37.1)
Yes	327 (62.9)
Having a close relative who had breast cancer made me do BSE (Cues 4)	
No	295 (56.7)
Yes	225 (43.3)

Note: RM = Ringgit Malaysia.

**Table 3.** Summary of fit indices of the path models.

Model	Fit indices				
	$\chi_{diff}^{2}$(df), *p*-value	TLI	CFI	RMSEA (90% CI)	SRMR
Model 1	-	0.106	0.461	0.113 (90% CI: 0.096, 0.130)	0.033
Model 2	-	−0.767	0.939	0.071 (90% CI: 0.137, 0.107)	0.020
Model 3 (Final)	206.455, df = 9, *p*-value < 0.001	0.845	0.945	0.062 (90% CI: 0.030, 0.096)	0.020

Note: χ^2^ = Chi-square; df = degree of freedom; χ^2^_diff_ = Chi-square difference; TLI = Tucker-Lewis Index; CFI = Comparative Fit Index; GFI = Goodness of fit index; RMSEA = Root Mean Square Error of Approximation; SRMR = Standardized Root Mean Square Residual; CI = confidence interval.

**Figure 2.**
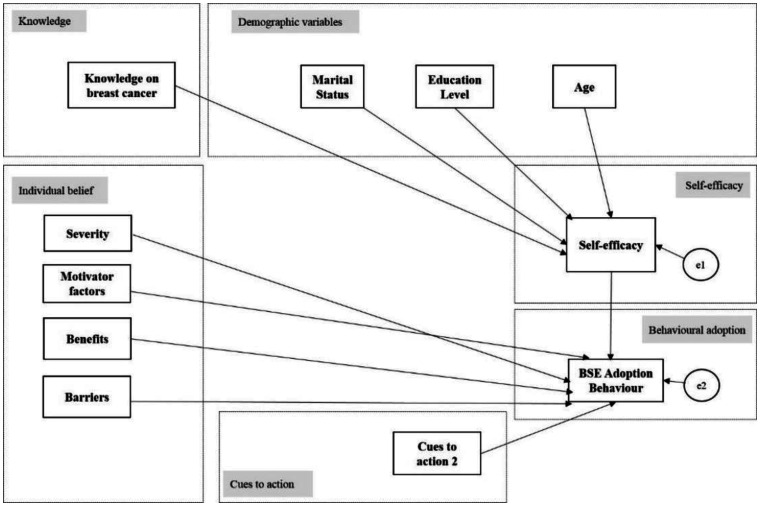
Model 2 path model after removing non-significant paths. Note: e1–e2 are the errors in measurement.

Non-significant paths between variables (standardized parameter estimates, β) that did not explain much of the model were removed iteratively. The model was then re-tested and evaluated for fitness repeatedly. The non-significant paths, which were removed, were pathways linking socio-demographic factors (occupation, race, and family income) and self-efficacy. Further, the pathways that link individual beliefs to the behavioral adoption of BSE and cues to action to the behavioral adoption of BSE were also excluded. This resulted in the revised model (Model 2) for significant paths as depicted in Figure 2.

### 3.1. Structural model (Model 2) after excluding insignificant variables

Model 2 as shown in Figure 2 was obtained after several re-test and fitness evaluations with the exclusion of insignificant variables. As such, the path relationships were reduced to 10 paths and the model was examined for the goodness of fit. The fit indices indicated that the model was still not within the acceptable range of the recommended threshold values.

### 3.2. Structural model (Model 3) after addition of significant path variables and further removing the non-significant path

Modification index (MI) suggested that additional path relationships should be added to improve the model fitness. Adequate theoretical support was carried out to investigate the path relationships suggested through MI. As such, the path from motivator factors to self-efficacy was added to the model. Subsequently, additional paths from cues to action to self-efficacy, barriers to self-efficacy, and knowledge of breast cancer to the behavioral adoption of BSE were included. Model 3 was finally obtained after deletion of the non-significant path of education level and self-efficacy and the inclusion of additional path from age to the behavioral adoption of BSE as suggested by MI.

**Figure 3.**
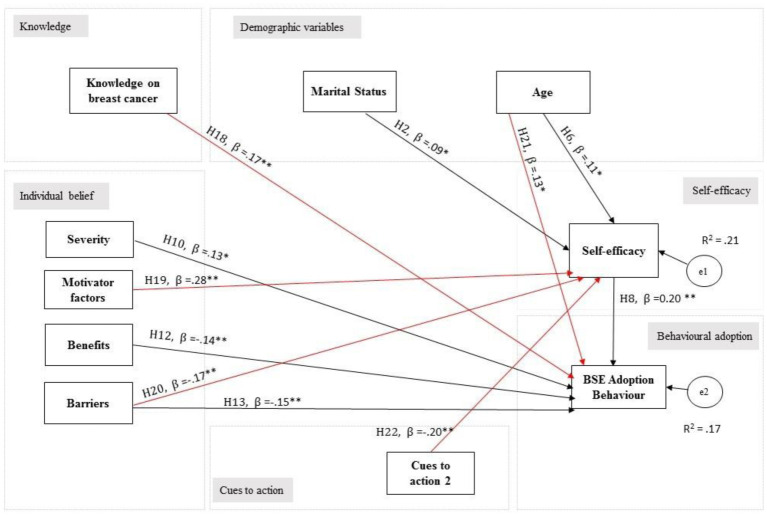
Model 3 (final model) path model after additional of significant paths. Note: e1–e2 are the errors in measurement, H = hypothesis; *p-value < 0.05; **p-value < 0.001; line in red = additional path added into the final model.

The model fitness shown in Table 3 indicated that the majority of the fit tests were within the acceptable range except for TLI with a value of 0.845 was slightly below the threshold value of marginal fit of 0.90. Figure 3 (Model 3) illustrates the theoretically important and significant relationships among the variables hypothesized.

### 3.3. Summary of the structural model testing and models' fit indices

In summary, Model 3 was accepted as the parsimonious model achieved after the addition of significant paths among the variables as suggested by MI. The $\Delta \chi_{MLR}^{2}$ was statistically significant *χ*^2^ as follows: $\Delta \chi_{MLR}^{2}$ = 206.455; df = 9; *p*-value < 0.001. Although $\Delta \chi_{MLR}^{2}$ was significant but the majority of the fit indices suggested a good fit of the proposed structural model (Model 3) to the observed data.

**Table 4.** Decision for the hypotheses based on Model 3 (Final model).

Hypothesis		Decision
H1:	Race significantly influence self-efficacy.	NS
H2:	Marital status significantly influence self-efficacy.	S
H3:	Education level significantly influence self-efficacy.	NS
H4:	Occupation significantly influence self-efficacy.	NS
H5:	Family income significantly influence self-efficacy.	NS
H6:	Age significantly influence self-efficacy.	S
H7:	Knowledge of breast cancer significantly influence self-efficacy.	NS
H8:	Self-efficacy significantly influence behavioral adoption of BSE.	S
H9:	Perceived susceptibility significantly influence behavioral adoption of BSE.	NS
H10:	Perceived severity significantly influence behavioral adoption of BSE.	S
H11:	Motivator factors significantly influence behavioral adoption of BSE.	NS
H12:	Perceived benefits significantly influence behavioral adoption of BSE.	S
H13:	Perceived barriers significantly influence behavioral adoption of BSE.	S
H14:	Cues to action 1 significantly influence behavioral adoption of BSE.	NS
H15:	Cues to action 2 significantly influence behavioral adoption of BSE.	NS
H16:	Cues to action 3 significantly influence behavioral adoption of BSE.	NS
H17:	Cues to action 4 significantly influence behavioral adoption of BSE.	NS
^a^H18:	Knowledge significantly influence behavioral adoption of BSE.	S
^a^H19:	Motivation factors significantly influence self-efficacy.	S
^a^H20:	Cues to action 2 significantly influence self-efficacy.	S
^a^H21:	Age significantly influence behavioral adoption of BSE.	S
^a^H22:	Perceived barriers significantly influence self-efficacy.	S

Note: H = hypothesis; NS = Not Supported; S = Supported; ^a^H = additional path.

Table 4 summarizes the hypotheses tested using SEM for the structural modeling of the study. Out of 17 paths tested, only six hypotheses were supported with five additional paths. Table 5 shows the significant regression weights (standardized path estimates) and path relationships between variables for the final model (Figure 3). Individual β, 95% CI, standard errors and the *p*-value for the 11 paths were presented in Table 5. The results display in Figure 3, indicated that the hypothesized model explained a statistical amount of variance for each latent variable. The overall model explained only 17% of the variance in behavioral adoption for BSE and 21% in self-efficacy.

**Table 5.** Path relationships of Model 3.

H	Relationships			β (95% CI)	SE	*p*-value
H2	Self-efficacy	←	Marital status (married)	0.089 (0.017, 0.160)	0.036	0.015
H6	Self-efficacy	←	Age (46–55 years old)	0.114 (0.040, 0.188)	0.038	0.002
H8	BSE behavior	←	Self-efficacy	0.199 (0.120, 0.278)	0.040	<0.001
H10	BSE behavior	←	Perceived severity	0.134 (0.048, 0.221)	0.044	0.002
H12	BSE behavior	←	Perceived benefits	−0.143 (−0.226, −0.060)	0.042	0.001
H13	BSE behavior	←	Perceived barriers	−0.149 (−0.225, −0.073)	0.039	<0.001
^a^H18	BSE behavior	←	Knowledge	0.173 (0.092, 0.253)	0.041	<0.001
^a^H19	Self-efficacy	←	Motivator factors	0.278 (0.208, 0.348)	0.036	<0.001
^a^H20	Self-efficacy	←	Cues to action	−0.200 (−0.274, −0.125)	0.038	<0.001
^a^H21	BSE behavior	←	Age (46–55 years old)	0.135 (0.064, 0.206)	0.036	<0.001
^a^H22	Self-efficacy	←	Perceived barriers	−0.169 (−0.255, −0.083)	0.044	<0.001

Note: H = hypothesis; β = standardized regression weights of pathways; SE = standard errors; *p*-value = probabilities value; CI = Confidence Interval; ^a^H = new path added into the model.

### 3.4. Moderation on the path model

The moderation effects of knowledge and socio-demographic factors (age and marital status) on the relationship of health beliefs (independent variables) and the behavioral adoption of BSE was conducted as the dependent variable. Summary of the interaction terms (psychologically meaningful) in the final Model 3 was depicted in Table 6.

**Table 6.** Moderation effect of knowledge and socio-demographic (age and marital status).

H	Relationship	β (95% CI)	SE	*p*-value
Knowledge	
H10	Perceived severity → Behavioral adoption of BSE	−0.081 (−0.163, 0.000)	0.002	0.051
H12	Perceived benefits → Behavioral adoption of BSE	−0.106 (−0.185, −0.028)	0.040	0.008
H13	Perceived barriers → Behavioral adoption of BSE	0.021 (−0.053, 0.095)	0.038	0.580
Age 45–55 years old	
H10	Perceived severity → Behavioral adoption of BSE	−0.048 (−0.118, 0.022)	0.036	0.183
H12	Perceived benefits → Behavioral adoption of BSE	−0.047 (−0.111, 0.016)	0.032	0.144
H13	Perceived barriers → Behavioral adoption of BSE	0.019 (−0.048, 0.086)	0.034	0.578
Marital status (married)	
H10	Perceived severity → Behavioral adoption of BSE	0.060 (−0.012, 0.166)	0.045	0.088
H12	Perceived benefits → Behavioral adoption of BSE	−0.125(−0.206, −0.044)	0.041	0.002
H13	Perceived barriers → Behavioral adoption of BSE	0.057(−0.021, 0.134)	0.040	0.152

Note: H = hypothesis; *p*-values marked in bold indicates significant moderation path.

Results from Table 6 indicated that knowledge and marital status moderate the relationship between perceived benefits and the behavioral adoption of BSE.

### 3.5. Testing self-efficacy as a mediator

**Table 7.** Standardized direct, total indirect and total effects.

Predictor Variables	Through	Causal effect		
		Direct	Indirect	Total
Knowledge → Behavioral adoption of BSE		
Knowledge	Self-efficacy	0.172	0.013	0.186
		*p*-value ≤ 0.001	*p*-value = 0.093	*p*-value ≤ 0.001
Age → Behavioral adoption of BSE		
Age	Self-efficacy	0.126	*0.022	0.147
(45–55 years old)		*p*-value ≤ 0.001	*p*-value = 0.010	*p*-value ≤ 0.001
Marital status → Behavioral adoption of BSE		
Marital status	Self-efficacy	0.070	*0.017	0.086
(married)		*p*-value = 0.122	*p*-value = 0.030	*p*-value = 0.059

Note: *Significant indirect relationship.

Table 7 indicated that self-efficacy mediates the relationship between age (45–55 years old) and the behavioral adoption of BSE (*p*-value = 0.010). Additionally, self-efficacy was also found to mediate the relationship between marital status (married) and the behavioral adoption of BSE (*p*-value = 0.030).

## 4. Discussion

The path relationships portrayed in the final model shows a significant influence of self-efficacy on the behavioral adoption of BSE. This is possibly due to self-efficacy being a significant predictor of behavioral intentions [27],[28] that actually precede actual behavior [29]–[31]. An individual that possesses vigorous self-efficacy would likely be engaged in taking defensive action in receiving information within a suitable time frame by promoting the likelihood of taking effective counteractive action [31]–[33]. Additionally, self-efficacy is believed to promote participants' confidence as studies found self-efficacy to be higher in women who competently [34]–[37] detect tumors successfully and correctly [38] using BSE. This is undeniable as women were more likely to perform BSE if they were confident in conducting the examination [12] as BSE is skilled orientated.

The findings showed that marital status (married women) and age (45–55 years old) significantly influence self-efficacy as married women often encounter lactation problems and also have high breast cancer risk due to their age. This could be a possible reason for their increased awareness to carry out BSE [39]. Further, women in this age group tend to be exposed to early breast cancer detection activities in healthcare settings such as BSE [40],[41]. Thus, they are likely to be more proactive in engaging in early breast cancer detection [41],[42]. Similarly, previous studies reported that married women were approximately five times more inclined to perform BSE than single women [27]. This could be due to spousal support that influences health-related behavior, such as in encouraging them to perform BSE [41],[43]. Additionally, older and menopause women are more likely to perform BSE as they perceived themselves as more susceptible to breast cancer [28]. This is an important finding from this study which explains why age has a direct influence on the behavioral adoption of BSE.

This study also reflected that motivator factors and perceived barriers have a significant relationship with BSE self-efficacy. This finding is consistent with previous research findings that women who perform BSE have higher levels of health motivation and as such, perceive higher self-efficacy in performing BSE [27]. Further, it was found that women who practiced BSE also have less perceived barriers [44]. However, the study indicated a negative correlation between cues to action (by talking with friends, partner or healthcare provider) and BSE self-efficacy. This could be due to individuals with positive health behaviors tend to think that negative events are less likely to happen to them compared to other people [45]. Hence, this optimism resulted in the negative correlation between cues to action that is by talking with friends, partner or healthcare provider did not result in BSE self-efficacy.

Perceived severity, perceived benefits and perceived barriers were also found to significantly influence the behavioral adoption of BSE. This is consistent with the HBM theory that women with high perceived severity of breast cancer will perceive BSE as beneficial which consequently led them to have low perceived barriers to perform regular BSE [28],[46]. Perceived severity is strongly related to sick-role behavior [47] and this is reflected in the findings of a previous study that women who perceived breast cancer as severe were reported to perform BSE 2.38 times more than women who do not [46]. The questions on perceived severity of breast cancer is as in Appendix II. Further, the finding of this study is in line with the HBM theory that perceived barriers in undertaking BSE may act as impediments in which an unconscious, cost-benefit analysis occurs in weighing perceived benefits versus barriers [47]. However, a negative correlation existed between perceived benefits and BSE adoption. Usually, results are interpreted using established theories or relevant literature to explore the relationships between constructs or variables. The negative correlation obtained between perceived benefits and BSE adoption could be the result of covariation rather than explanations. As most of the participants are Muslims, this might be because of perceived taboos about touching or exposing their body parts. They might avoid adopting BSE even though it is beneficial maybe because of fear of breast cancer diagnosis [48]. The questions used in the survey on perceived benefits of BSE is as in Appendix III.

Adequate knowledge of breast cancer was found to have a positive impact on the behavioral adoption of BSE [49]. This is in agreement with the finding of this study which showed a significant influence of knowledge on breast cancer on the behavioral adoption of BSE as indicated from the additional path of the model created. Similarly, a previous study also reported that women that have a high level of knowledge on breast cancer performed more frequent BSE compared to those with a low level of knowledge. This indicated that knowledge plays a vital role in the adoption of health-promoting behaviors [46]. Additionally, knowledge was also found to moderate the relationship between perceived benefits and the behavioral adoption of BSE. In this regard, women must have an adequate level of knowledge on breast cancer in order to define symptoms and risk factors of breast cancer to enable early detection of breast cancer using BSE [41],[50],[51]. Further, marital status was also found to moderate the relationship between perceived benefits and the behavioral adoption of BSE. Previous studies indicated that critical benefits of marriage include economic resources and social support [41],[52]–[54] which then possibly augmented the relationship between perceived benefits and behavioral adoption of BSE.

Additionally, self-efficacy was also found to mediate the relationship between age (45–55 years old) as well as marital status (married) with the behavioral adoption of BSE. This could possibly due to the fact that married women have the spousal economic and social support which motivates them to regularly perform BSE. On the contrary, it was found that women aged below 50 years old have higher positive attitudes in performing BSE and as such would incline to efficaciously perform the procedure [41],[55]. However, worry and anxiety about breast cancer risk amongst older women were identified as reasons for the poor performance of BSE [41],[56],[57]. According to the Social Cognitive Theory, for behavioral change to take place, self-efficacy is the initiator and also the core construct for the maintenance of behavioral change [58]. As such for behavior change to succeed, women must be self-efficacious in overcoming perceived barriers and believe that change is beneficial at an acceptable cost.

## 5. Limitations

There are several limitations to this study. First, due to the employment of cross-sectional design for this study, inference on the influence of causal relationships amongst variables cannot be determined. Second, the data were collected based on the self-report measures that could lead to response bias in participants in reporting their BSE behavior and practices. Third, although path analysis is to test how well the hypothesized model fits the data or the process behind a phenomenon of interest, the researchers cannot infer that the model best represents the phenomenon [59]. Finally, the model cannot be generalized to all women in Malaysia on the adoptive behavior of BSE as the data was collected from women living in Kuantan, Pahang. This is because the socio-demographic makeup such as urbanization level and racial makeup are different compared to other states in Malaysia. This in turn affects their education, technology, hospital care and cultural beliefs.

## 6. Conclusion

This study found perceived severity, perceived benefits and perceived barriers to significantly influence the behavioral adoption of BSE which is consistent with the HBM theory that women with high perceived severity of breast cancer will perceive BSE as beneficial which consequently led them to have a low perceived barrier to perform regular BSE. Self-efficacy is the core construct found in the study to influence the behavioral adoption of BSE. This is undeniable as self-efficacy is believed to promote participants' confidence. However, only knowledge and marital status (married) were found to moderate between perceived benefits and the behavioral adoption of BSE whilst self-efficacy mediates the relationship between age and marital status (married) with the behavioral adoption of BSE. This is in accordance with the Social Cognitive Theory that for a change of behavior to take place, self-efficacy serves as the initiator and maintenance of behavioral change. As such for a behavior change to succeed, women must be self-efficacious in overcoming impediments and believe that change is beneficial at an acceptable outcome.
