# Supplementary material for: A path analytic model of health beliefs on the behavioral adoption of breast self-examination
Source: AIMS Public Health. 2020 Dec 21;8(1):15–31. doi: 10.3934/publichealth.2021002 (PMC7870382; doi:10.3934/publichealth.2021002)
Supplement: Supplementary file 1 [file publichealth-08-01-002-s001.pdf]

AIMS Public Health
AIMS Public Health, 8(1): 15–31.
DOI: 10.3934/publichealth.2021002
Received: 07 November 2020
Accepted: 17 December 2020
Published: 21 December 2020
http://www.aimspress.com/journal/aimsph

Research article

A path analytic model of health beliefs on the behavioral adoption of breast self-examination

Soo-Foon Moey[1,*], Norfariha Che Mohamed[1] and Bee-Chiu Lim[2]

[1] Department of Diagnostic Imaging and Radiotherapy, Kulliyyah of Allied Health Sciences, International Islamic University Malaysia (IIUM), Kuantan Campus, Pahang, Malaysia
[2] Clinical Research Centre, Hospital Tengku Ampuan Afzan (HTAA), Kuantan, Pahang, Malaysia

* **Correspondence:** Email: moeysf@iium.edu.my; Tel: +60127751439.

Appendix I

Cues to action

Strongly disagree (Sangat tidak bersetuju) → Strongly agree (Sangat bersetuju)

	1	2	3	4	5	6	7	8	9	10
1. I have heard about BSE from the mass media: books/magazines/pamphlets/health education materials/television or video/*Saya mengetahui tentang pemeriksaan sendiri payudara melalui media massa seperti; buku/majalah/pamplet/material kesihatan/ televisyen atau radio*										
2. I have heard about BSE from talking with: friends/partner/doctor/healthcare provider/*Saya mengetahui tentang pemeriksaan sendiri payudara melalui perbualan antara saya dan; kawan/suami/doktor/pegawai kesihatan*										
3. Having known someone who had breast cancer made me do BSE/*Mengenali seseorang yang menghidapi kanser payudara membuatkan saya melakukan pemeriksaan sendiri payudara*										
4. Having a close relative who had breast cancer made me do BSE/*Mempunyai ahli keluarga rapat yang menghidapi kanser payudara membuatkan saya melakukan pemeriksaan sendiri payudara*										

Appendix II

Perceived Severity on Breast Cancer

Strongly disagree → Strongly agree
(Sangat tidak bersetuju) (Sangat bersetuju)

	1	2	3	4	5	6	7	8	9	10
1. I think I will not live more than 5 years with breast cancer/*Saya percaya saya tidak akan hidup lebih dari 5 tahun jika saya menghidapi kanser payudara*										
2. When I think about breast cancer my heart beat faster/*Jantung saya berdegup laju apabila memikirkan tentang kanser payudara*										
3. I am afraid even to think about breast cancer/*Saya merasa takut memikirkan tentang kanser payudara*										
4. All my life will be changed if I got breast cancer/*Kehidupan saya akan berubah jika saya menghidapi kanser payudara*										
5. The thought of breast cancer scares me/*Apabila saya memikirkan tentang kanser payudara, ia menakutkan saya*										

Appendix III

Perceived Benefits of BSE

Strongly disagree → Strongly agree
(Sangat tidak bersetuju) (Sangat bersetuju)

	1	2	3	4	5	6	7	8	9	10
1. Performing BSE monthly help in early detection of breast cancer/*Menjalankan pemeriksaan sendiri payudara secara kadar bulanan dapat membantu dalam pengesanan awal kanser payudara*										
2. Performing BSE monthly help in detection of tumors before going to the doctors/*Menjalankan pemeriksaan sendiri payudara secara kadar bulanan dapat membantu dalam pengesanan awal kanser payudara sebelum berjumpa doktor*										
3. Performing BSE monthly will decrease complications of breast cancer if I got breast cancer/*Menjalankan pemeriksaan sendiri payudara secara kadar bulanan boleh mengurangkan komplikasi terhadap kanser payudara sekiranya saya menghidapinya*										
4. Performing BSE decrease the chance of surgery if I got breast cancer/*Menjalankan pemeriksaan sendiri payudara dapat mengurangkan kemungkinan pembedahan sekiranya saya menghidap kanser payudara*										
5. Performing BSE decrease the anxiety about breast cancer/*Menjalankan pemeriksaan sendiri payudara dapat mengurangkan keresahan tentang kanser payudara*										

AIMS Press

© 2021 the Author(s), licensee AIMS Press. This is an open access article distributed under the terms of the Creative Commons Attribution License (http://creativecommons.org/licenses/by/4.0)
